# High-resolution T2 relaxometry detects intramyocardial hemorrhage post myocardial infarction in swine

**DOI:** 10.1186/1532-429X-16-S1-O71

**Published:** 2014-01-16

**Authors:** Haiyan Ding, Karl H Schuleri, Michael Schär, M Muz Zviman, Roy Beinart, Daniel A Herzka

**Affiliations:** 1 Biomedical Engineering, Tsinghua University, Beijing, China; 2 Biomedical Engineering, Johns Hopkins School of Medicine, Baltimore, Maryland, USA; 3 Philips Healthcare, Cleveland, Ohio, USA; 4 Medicine, Cardiology, Johns Hopkins School of Medicine, Baltimore, Maryland, USA; 5 Heart Institute, Sheba Medical Center, Tel Aviv University, Ramat Gan, Israel

## Background

Intramyocardial hemorrhage (IMH) secondary to myocardial infarction (MI) is associated with adverse prognosis [1]. The byproducts of blood breakdown lead to decreases in T2 and T2*, and have been shown to be related to microvascular obstruction (MVO) as detected by early contrast enhanced (EGE) imaging [2]. However, EGE is heavily influenced by contrast agent kinetics and measures the lack of contrast agent penetrance rather than IMH directly. Here, we present high-resolution T2 mapping for accurate assessment of IMH without the need for contrast agents. Hypothesis: Quantitative high resolution T2 mapping can detect IMH, without the need for contrast agents.

## Methods

MI was induced by 120 min LAD occlusion and reperfusion in 5 Yorkshire swine. Imaging was performed 5-9 days post MI using a 3T system (Achieva TX, Philips Healthcare). Breath-hold black-blood T2W turbo spin echo imaging (BB-T2-STIR) [3] and 3D respiratory navigator gated T2-mapping [4] were acquired. EGE images (phase sensitive inversion recovery [5]) were also acquired post infusion (0.2 mmol/kg, Magnevist). 3D T2 maps were calculated per voxel using linear regression of the log of the signal and poor fits (R2 < 0.9) were rejected. IMH was identified in T2W images/T2 maps by areas of hypointense signal/T2 surrounded by hyperintense signal/T2 representing edema. MVO was defined in EGE images by a hypointense area surrounded by enhanced MI. After MRI acquisitions hearts were excised and post-mortem pathology and histology were obtained.

## Results

**Figure 1:**
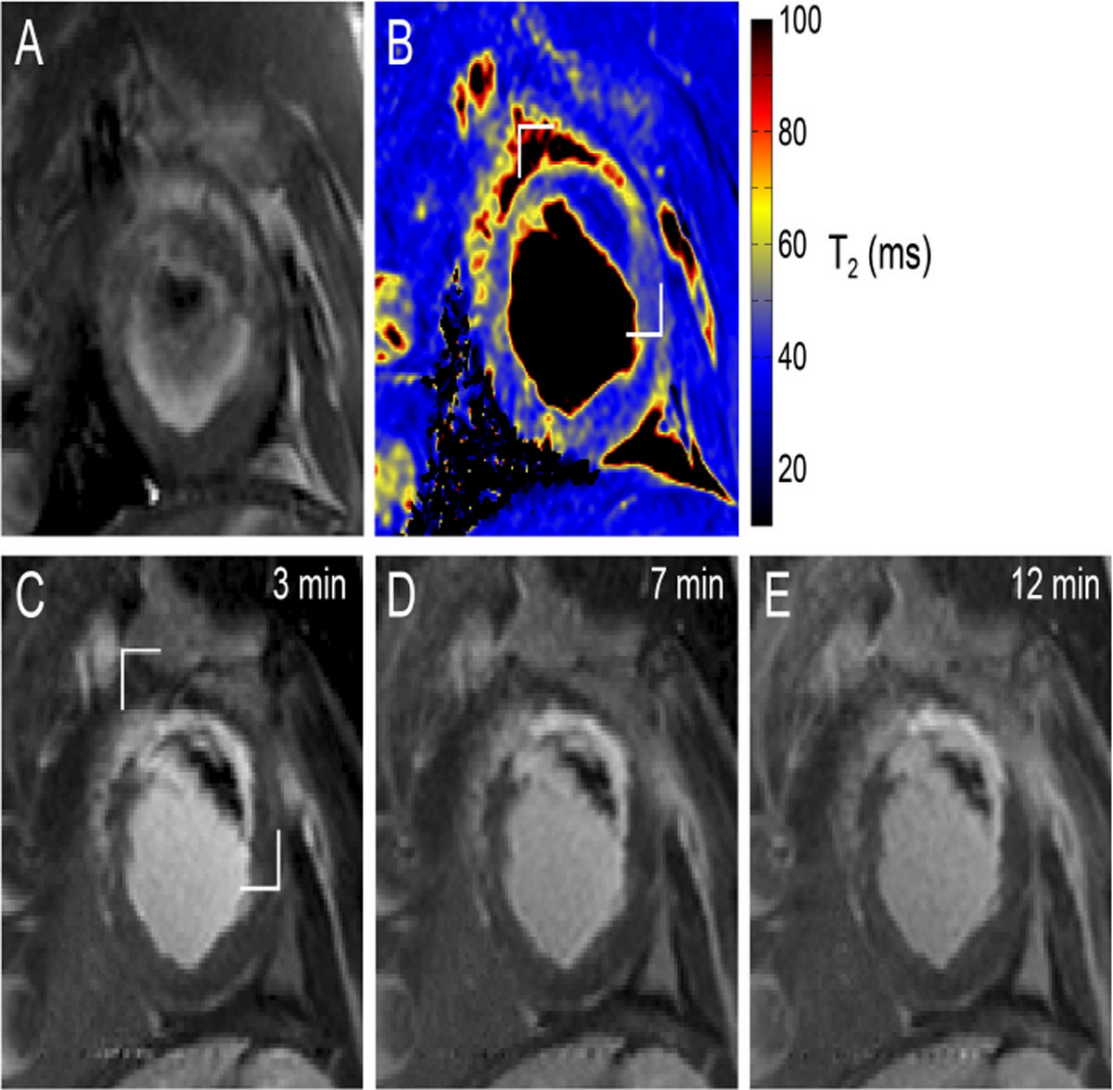
**Matched representative SAX slices from in vivo pre contrast T2W (BB-T2-STIR) (A) and pre-contrast T2 map (B)**. A time series of post contrast EGE (PSIR) are shown in (C-E) with time post infusion shown. Significant T2 reduction with heterogeneity is presented from the T2 map (magnified in Figure 2). MVO is clearly detected from EGE as the hypointensity surrounded by enhanced signal though the temporal variation of the area of MVO represents the sensitivity to contrast agent kinetics.

**Figure 2:**
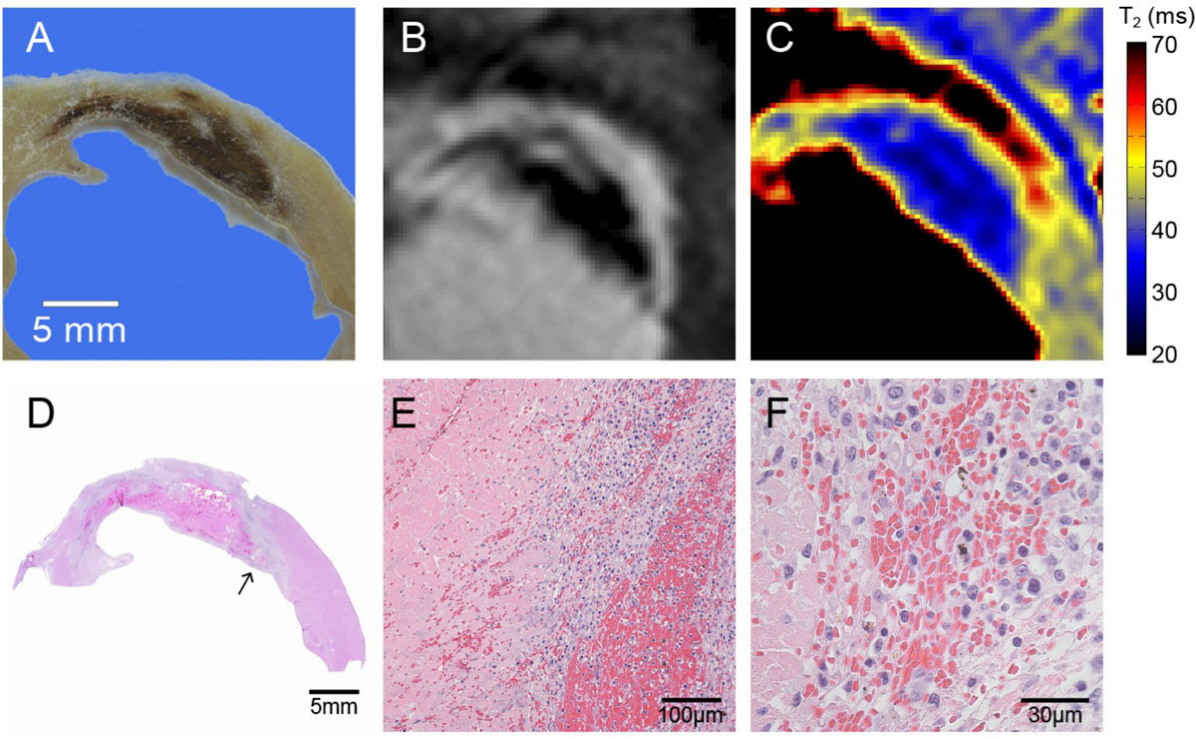
**Matched representative SAX slices from ex vivo (A) and in vivo 3 min post contrast EGE (B) and pre-contrast T2 map (C)**. Gross pathology from the fixed ex vivo heart shows a dark hemorrhagic zone (A). Magnified versions of (Fig 1 C) and (Fig 1 B) are shown in (B) and (C). Significant T2 reduction with heterogeneity is presented from the T2 map. The shape and extent of MVO/hemorrhage from both EGE and T2 map are highly consistent with that from gross pathology (A). (D-F) Hematoxylin and eosin staining depicts viable myocardium in pink from necrotic tissue in light blue and IMH in dark pink or red. At higher magnification the densely hemorrhagic core (E) and necrosis with infiltrated blood cells (F) can be seen.

IMH was detected in 4 (out of 5) animals by T2 mapping which was confirmed by histology. Findings on T2W images were variable due to prominent artifacts from slow flow or motion, though signal heterogeneity was observed (Figure 1). MVO was detected from EGE though MVO varied with time post contrast injection. T2 mapping showed excellent correlation with the myocardial distribution of IMH as evidenced by reduced T2 and the correlation with MVO. (Figure 2) Based on ROI analysis, T2 in IMH was significantly lower than that of remote myocardium (36.5 ± 4.6 ms v.s. 45.7 ± 1.1 ms, p = 0.02).

## Conclusions

High-resolution T2 imaging allows for accurate assessment of IMH without the temporal variability imposed by the contrast agent kinetics of EGE, or the known artifacts associated with BB-T2-STIR.

## Funding

This work was funded in part by AHA-11SDG5280025.
